# Electrical Mobility as an Indicator for Flexibly Deducing the Kinetics of Nanoparticle Evaporation

**DOI:** 10.1021/acs.jpcc.2c02858

**Published:** 2022-05-11

**Authors:** Huan Yang, Dian Ding, Aurora Skyttä, Runlong Cai, Markku Kulmala, Juha Kangasluoma

**Affiliations:** Institute for Atmospheric and Earth System Research/Physics, University of Helsinki, FI-00014 Helsinki, Finland

## Abstract

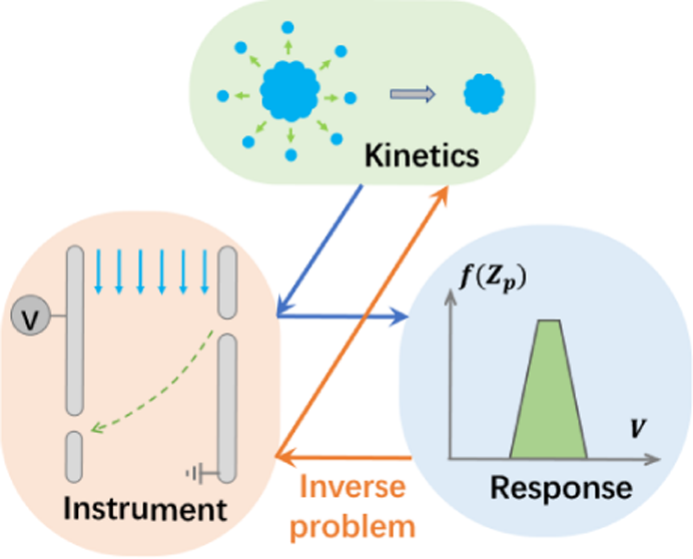

Condensation and evaporation of vapor species on nanoparticle surfaces drive the aerosol evolution in various industrial/atmospheric systems, but probing these transient processes is challenging due to related time and length scales. Herein, we present a novel methodology for deducing nanoparticle evaporation kinetics using electrical mobility as a natural size indicator. Monodispersed nanoparticles are fed to a differential mobility analyzer which serves simultaneously as an evaporation flowtube and an instrument for measuring the electrical mobility, realizing measurements of evaporation processes with time scales comparable to the instrument response time. A theoretical framework is derived for deducing the evaporation kinetics from instrument responses through analyzing the nanoparticle trajectory and size–mobility relationship, which considers the coupled mass and heat transfer effect and is applicable to the whole Knudsen number range. The methodology is demonstrated against evaporation but can potentially be extended to condensation and other industrial/atmospheric processes involving rapid size change of nanoparticles.

## Introduction

The formation and growth of nanometer-scale particles from vapor precursors^1,2^ are critical in a diverse array of aerosol systems,^3,4^ with the atmospheric new particle formation^5^ (NPF) being an important example. For continuous nanoparticle formation and growth, condensation (i.e., the acquisition of vapor molecules) serves as the driving force, while evaporation (i.e., the loss of vapor molecules) sets the barrier. Probing these dynamic processes and quantifying their net effects on nanoparticle growth are hence of central importance. A common requirement for probing dynamic processes is to ensure that the response time of the instrument is much smaller than the time scale of the process itself. Current state-of-the-art techniques for probing nanoparticle dynamics, such as the electrical mobility spectrometers^6,7^ (EMSs), are typically limited to a response time of around a few seconds. Thus, the application of these instruments has been limited to equilibrium processes^8−10^ or dynamic processes with comparatively large time scales.^11,12^ Probing very rapid dynamic processes has been done only on molecular ions with up to tens of molecules,^13−16^ with one exception by Wright et al.,^17^ where submicro scale particles were measured. A typical EMS system is composed of a particle charger, a differential mobility analyzer (DMA), and a particle detector. For a given voltage, the DMA only allows particles within a narrow neighbor of a specific electrical mobility to pass through, the number of which is then carefully counted by the downstream particle detector. As electrical mobilities of certain particles can be uniquely linked to their sizes with appropriate physical models^18,19^ after multiple charge correction, the response for EMS systems is thereby the number of particles at multiple discrete voltages/sizes in the measured voltage/size range. Due to the high sensitivity and robust performance, EMSs have been implemented in different research areas and have facilitated the understanding of various nanoparticle-related physical/chemical processes.^20−24^ However, so far, it is still challenging for such systems to investigate processes involving rapid nanoparticle size changes on sub-second time scales. Such processes are commonly encountered in atmospheric processes or engineered systems, where there are sudden changes in surrounding gas conditions^25,26^ and nanoparticles change their sizes rapidly to respond.

Due to the negligible inertia, nanoparticles can quickly adjust their equilibrium velocities to accommodate the change of sizes when migrating in the flow and electric field in the DMA. The transient velocity, or transient mobility, is hence a natural size indicator, with a response time comparable to the nanoparticle relaxation time in the airflow (e.g., ∼10^–7^ s for a 100 nm particle with the same density as water). By taking advantage of this fact, this work proposes a novel methodology, allowing for the measurement of rapid nanoparticle growth or shrinkage processes. The methodology is demonstrated with a conventional EMS system, where, however, the DMA is operated unconventionally: instead of maintaining a constant nanoparticle size, continuous size change of nanoparticles is intentionally induced during their migrations in the DMA sheath flow. In the example of evaporation, the change of nanoparticle size is achieved by placing the DMA in a thermal insulation chamber, where the temperature is elevated compared to the inlet aerosol flow. We reveal two critical requirements that the evaporating nanoparticle needs to satisfy. (1) To pass through the DMA, the time average transient mobility *Z*_p_(*t*) of the nanoparticle must be a constant: e.g., (∫_0_^*t*^*Z*_p_(*t*′) d*t*′)/*t* = Ω, where Ω is a constant dependent on the DMA settings in a measurement. (2) The evaporation process requires that the nanoparticle transient mobility follows some unique functional form: e.g., *Z*_p_(*t*) = *f*(*C*, *t*), where *f* is a known function that can be derived from the physical/chemical process and *C* is a lumped and unknown constant dependent on associated thermodynamic and kinetic parameters. The combination of the two requirements leads to the determination of the nanoparticle size-change history based on the EMS response without any prior knowledge about the thermodynamic and kinetic parameters.

## Methods

### Transmission of Size-Changing Nanoparticles in the DMA

**Figure 1:**
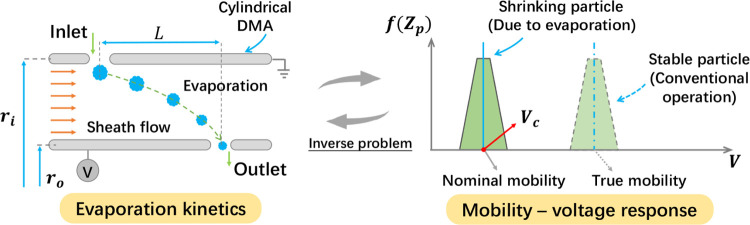
Schematic for deducing the nanoparticle evaporation kinetics from the device response: solving an inverse problem.

The first requirement on the size-changing nanoparticle results from the electric and flow field structure inside the classification region of the DMA. Suppose that a nanoparticle enters a cylindrical DMA^6^ from the inlet slit with a mobility of *Z*_pi_ and undergoes continuous evaporation before leaving the outlet slit with a mobility of *Z*_po_, as shown in Figure 1. The radial and axial motion of the nanoparticle are driven by the electrical field  and flow field , respectively, which are steady in time. With the inlet and outlet slits assumed to be infinitesimal, and particle diffusion and inertia neglected, the equations that govern the transmission of the nanoparticle are
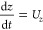
1a
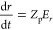
1bwhere *r* and *z* are respective radial and axial coordinates of the nanoparticle trajectory, *Z*_p_ = *Z*_p_(*t*) is the transient nanoparticle mobility, and *U_z_* and *E_r_* are positive if pointing in the same direction as the axis and negative otherwise. eq 1a is nothing but *L* = *U*_*z*_*t*_f_ after integration, where *L* is the axial distance between the inlet and outlet slit, and *t*_f_ is the nanoparticle residence time. Because of the cylindrical geometry, the electrical field is dependent on the radial coordinate, i.e., *rE*_*r*_ = *V*/ln (*r*_i_/*r*_o_), where *V* is the voltage applied to the inner rod of the cylindrical DMA (its outer shell is grounded), and *r*_i_ and *r*_o_ are the radial coordinates of the inlet and outlet slit, respectively. Substituting the expression of *E*_*r*_ into eq 1b and integrating, one obtains (*r*_o_^2^ – *r*_i_^2^)/2 = *V*/ln(*r*_i_/*r*_o_)∫_0_^*t*_f_^* Z*_p_ d*t*, which can be rearranged to achieve
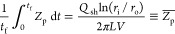
2where *Q*_sh_ = π(*r*_i_^2^ – *r*_o_^2^)*U*_*z*_ is the sheath flow rate and  is a constant defined as the “nominal mobility”, which depends on the DMA geometric parameters, sheath flow rates, and voltage. Note that we have dropped a negative sign in eq 2 for simplicity. Equation 2 states that to pass through the DMA, the time average mobility of the size-changing nanoparticle must be a constant if the DMA settings are fixed. For conventional operations, where nanoparticles do not change size, eq 2 uniquely links the constant nanoparticle mobility *Z*_p_ with a DMA voltage. However, due to non-negligible aerosol flow rates, realistic device responses are trapezium-shaped functions (the so-called transfer function, as shown in Figure 1), even if nanoparticles are monodispersed. Therefore, there is no one-to-one mapping between the DMA voltage and nanoparticle mobility *Z*_p_. A strict discussion on nanoparticle transmissions with transient mobilities needs also be done under the framework of the transfer function^6,27,28^ that explains the shape of the device response, and this will be addressed in detail in future work. Nonetheless, eq 2 has good accuracy for cases where aerosol inlet and outlet flows are small compared to the sheath flow, and it is essentially the requirement that the nanoparticle must satisfy to pass through the DMA.

### Temporal Mobility of Evaporating Nanoparticles

The physical process like evaporation or condensation requires that the nanoparticle transient mobility follows some unique functional form. To derive an expression for the function, we resort to the equations governing the size change of the evaporating nanoparticle
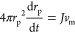
3where *r*_p_ is the radius of the nanoparticle, *v*_m_ is the volume of the (condensable) vapor monomer, and *J* is the evaporation flux. Depending on the Knudsen number (*K*_*n*v_ = λ_v_/*r*_p_, where λ_v_ is the vapor mean free path), the evaporation flux can be modeled under the framework of the kinetic gas theory, the Fick’s law of diffusion, or semi-empirical models like the Fuchs flux matching theory, corresponding to mass transport in the free molecular, continuum, or transition regime. These have been studied extensively in the literature.^29,30^ Here, we use the transition regime transport as an example and derivations/results for the other two regimes are provided in the Supporting Information. The evaporation flux in the transition regime is
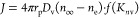
4awhere
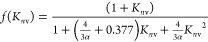
4bis a Knudsen number-dependent correction function,^31^ α is the mass accommodation/evaporation coefficient, *D*_v_ is the vapor monomer diffusion coefficient, *n*_∞_ is the vapor number concentration in the background gas (i.e., in the DMA sheath flow), and *n*_e_ is the equilibrium vapor number concentration at the nanoparticle surface, evaluated based on the Kelvin effect
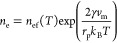
4cwhere *n*_ef_(*T*) is the flat surface equilibrium vapor number concentration evaluated based on corresponding vapor pressure *P*_ef_(*T*) (CHERIC, 2015, see Eq. SI 3.1 of Wright et al.^17^ for the expression), *k*_B_ is the Boltzmann constant, *T* is the temperature at the nanoparticle surface, and γ is the nanoparticle surface tension. Equations 4a–4c and 3 govern the temporal nanoparticle radius under isothermal conditions. However, evaporating nanoparticles can have decreased surface temperatures compared to the background gas due to loss of latent heat, which in turn decreases the rate of evaporation. Therefore, eq 4a needs to be corrected in cases where the latent heat loss rate is large. Kulmala et al.^32,33^ solved the coupled heat and mass transfer problem in the continuum regime considering decreased nanoparticle surface temperatures. Here, we apply the Knudsen correction to their results for evaporation flux in the transition regime, i.e.
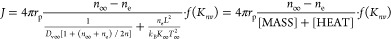
4dwhere *n* is the total concentration of vapor and gas in the background, *L* is the latent heat (J/#), *K*_∞_ is the background gas thermal conductivity, *T*_∞_ is the background gas temperature, and *D*_v∞_ is the vapor monomer diffusion coefficient at *T*_∞_. The first and second terms in the denominator characterize the influence of mass and heat transfer, respectively. In the Supporting Information (Figure S1), evaporations with and without heat transfer for free glycerol and water particles in air are compared. The effect of heat transfer is negligible in the case of glycerol but is significant in the case of water; hence, eq 4a is adopted in the subsequent discussion of glycerol particles. The implementation of eq 4d with non-negligible heat transfer effects can follow a similar procedure.

Our first goal is to get the equation of temporal nanoparticle mobility to assist eq 2; we thereby link the nanoparticle mobility to the radius through
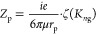
5awhere *i* is the number of charges on the nanoparticle, *e* is the elementary charge, *μ* is the background gas viscosity, and
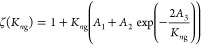
5bis the Cunningham correction factor^34,35^ with *A*_1_ = 1.257, *A*_2_ = 0.400, *A*_3_ = 0.55, and *K*_*n*g_ = λ_g_/*r*_p_, with λ_g_ being the background gas mean free path. Now, we can express *r*_p_ in terms of *Z*_p_ based on eqs 5a and 5b, which can then be substituted into eq 3 to obtain the equation for the temporal nanoparticle mobility
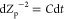
6awith
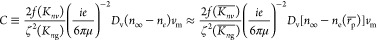
6bOriginally, the variable *C* in the above equations is not a constant because *K*_*nv*_, *K*_*n*g_, and *n*_e_ are nanoparticle radius-dependent, but we have made the following approximations:  and , where  and  are corresponding vapor and background gas Knudsen numbers evaluated at , and  is some sort of averaging of the nanoparticle radius when migrating in the device. It should be noted that though the actual choice of  will not affect the deduced temporal and sizes at the DMA outlet (e.g., eqs 7and 8a), these approximations alter the functional dependence of temporal size on time and hence introduce inherent errors in the theory. It can be shown that such errors are negligible in the studied size and temperature ranges (see Supporting Information Figure S2). The solution of eq 6a is readily available
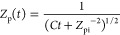
7The above equation is the form of function that the temporal nanoparticle mobility needs to follow due to the evaporation in the transition regime.

Substituting eq 7 into eq 2, we obtain the constant *C* and the corresponding mobility at the outlet
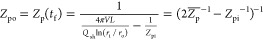
8a
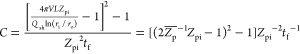
8bThis solution turns out to be extremely concise: the nanoparticle mobility at the DMA outlet *Z*_po_ is expressed by its mobility at the DMA inlet *Z*_pi_ and its nominal mobility  defined by eq 2 in terms of the geometric parameters, sheath flow rate, and voltage, while the nanoparticle temporal mobility (eq 7) can be determined based on the constant *C*, which is expressed by its mobility at the DMA inlet *Z*_pi_, its nominal mobility , and its residence time *t*_f_. Alternatively, nanoparticle trajectories and temporal sizes can be solved by numerical approaches. In such simulations, the associated thermodynamic and kinetic parameters are assigned/adjusted such that the simulated device response matches that of the measurement. The final set of parameters may be used to approximate the real measurement condition. Involved thermodynamic and kinetic parameters include the vapor pressure, surface tension, diffusion coefficient, mass accommodation coefficient, etc. A major difficulty faced by such a numerical approach is that there could be multiple combinations of free parameters leading to the agreement with the measured device response, but the uncertainties arising from selecting one of the possible combinations cannot be quantified. The requirements (e.g., eqs 2 and 7) revealed in this work allow the nanoparticle temporal mobility/size profile to be determined based on the EMS response without the need to know/assume any exact values of the thermodynamic and kinetic parameters, and hence avoid the associated uncertainties.

## Results and Discussion

### **Nanoparticle** Temporal Sizes and Sizes at the DMA Outlet

**Figure 2:**
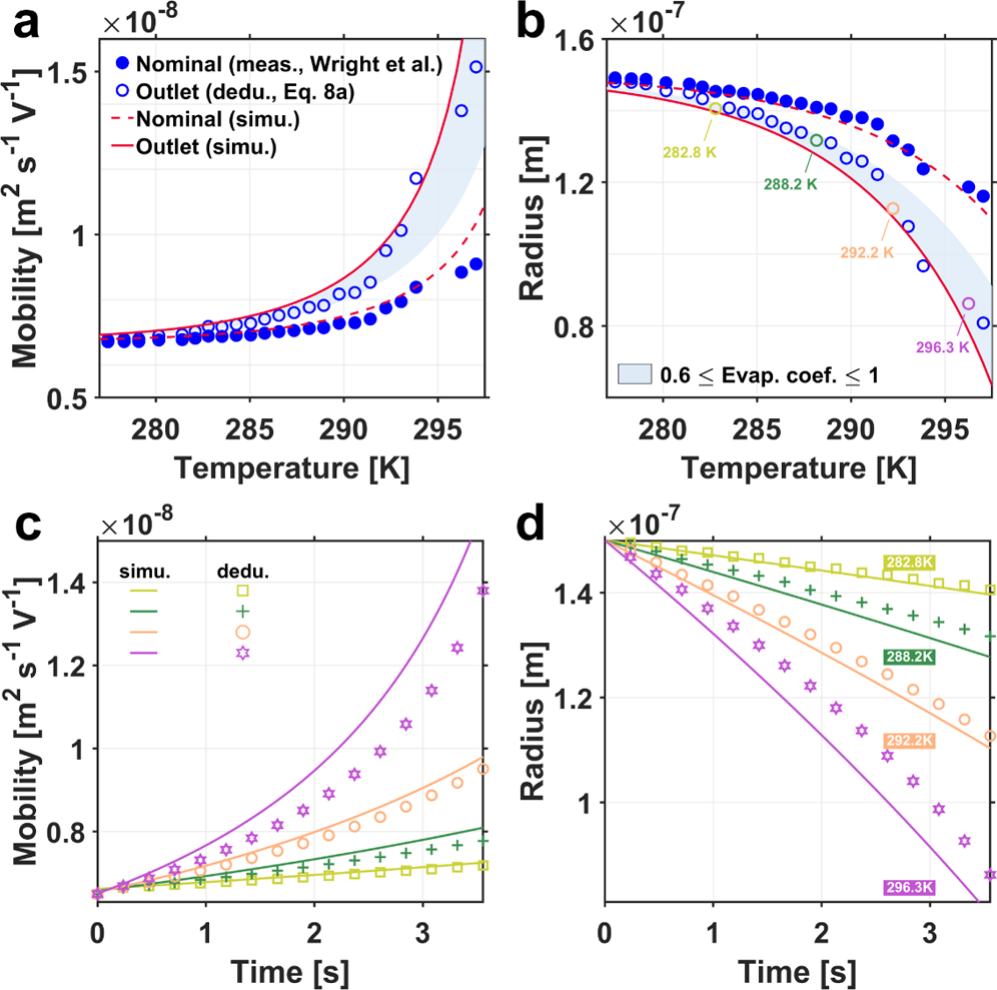
(a, b) measured nanoparticle nominal sizes (solid circles), deduced nanoparticle sizes at the DMA outlet (hollow circles, deduced based on eq 8a), simulated nanoparticle nominal sizes (dotted lines), and simulated nanoparticle sizes at the DMA outlet for the case of *Q*_sh_ = 26.48 L min^–1^ and *r*_pi_ = 151 nm: mobility (a) and radius (b), where the shadowed regions represent the simulated sizes at the DMA outlet with varying evaporation coefficients from 0.6 to 1. (c, d) simulated (solid lines) and deduced (dots, deduced based on eqs 7 and 8a) nanoparticle temporal mobility and radius profiles at selected system temperatures from (a) and (b): mobility (c) and radius (d).

Wright et al.^17^ measured the evaporation of glycerol nanoparticles inside the classification region of a cylindrical DMA under dry (*n*_∞_ = 0) and humid sheath flow conditions. They also solved the nanoparticle trajectories and transient sizes to compare with the experimental data such that several thermodynamic and kinetic parameters were deduced. A direct way to validate the current methodology is to check whether the transient predicted nanoparticle mobility *Z*_p_(*t*) or nanoparticle mobility at the outlet of the DMA *Z*_po_ matches the experimental data. However, as *Z*_p_(*t*) and *Z*_po_ are unknown in the experiments, we opt to validate the methodology by comparing the prediction of eqs 7 and 8a (using the experimentally measured nominal mobility  as the input) with numerical simulations. Figure 2a shows the measured glycerol nanoparticle nominal mobilities (Wright et al.,^17^ solid circles) and the deduced glycerol nanoparticle mobilities at the DMA outlet (eq 8a, hollow circles) at various system temperatures, with *Q*_sh_ = 26.48 L min^–1^ and *r*_pi_ = 151 nm. The results are compared with those obtained from numerical simulations based on the evaporation of a free glycerol nanoparticle, with α = 1, *n*_∞_ = 0, and the total simulation time *t* = *t*_f_ (see the Supporting Information for details of simulations). Good agreements can be observed with two major implications. (1) The agreement between the measured and simulated nominal mobilities indicates that the simulation can accurately describe the glycerol nanoparticle evaporation in the experiment and hence can be used to validate our methodology, and (2) the agreement between the deduced and simulated mobilities at the DMA outlet validates eq. 8 and indicates the feasibility of our methodology. More comparisons at different operation conditions are shown in Supporting Information Figure S3. We checked the influence of the evaporation coefficient α by varying its value from 0.6 to 1, as shown by the shadowed regions in Figure 2a,b. It is found that the deduced mobilities and radii agree better with the simulated results as α → 1, indicating that α = 1 is a reasonable assumption for particles at these sizes. An interesting feature of the current approach is that it deduces the temporal radius and mobility profiles of the evaporating nanoparticle from the measured nominal mobility (Figure 2c,d). Compared with simulated values, deviations are overall small at low temperatures but increase at high temperatures, especially in terms of the curvature of the temporal profiles. This is because the transient nanoparticle radius was approximated with an averaged radius in eq 6b, which alters the functional dependence of the temporal profile on the radius, and the accuracy of this assumption is expected to decrease as the size change of nanoparticles (from the DMA inlet to the outlet) increases. Comparisons between the simulated and deduced temporal evaporation fluxes are summarized in Supporting Information Figure S4, where similar trends in the deviations in curvature are observed. Overall, when applying this methodology to deduce temporal size or flux profiles, attention needs to be paid to balancing the accuracy and the investigated temperature range.

### **Flat Surface** Vapor Pressures

**Figure 3:**
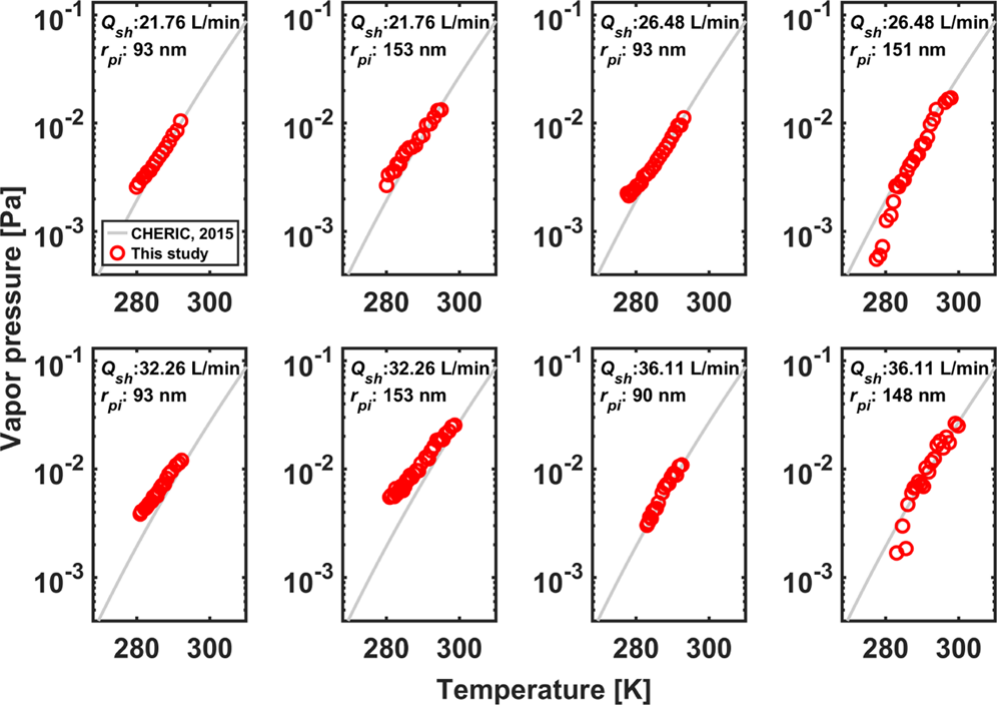
Deduced flat surface vapor pressure of glycerol from various operation conditions based on the proposed methodology.

The approach can be applied to estimate the flat surface vapor pressure based on the change of the nanoparticle radius from the DMA inlet to the outlet. Substituting eqs 4a and 4c into eq 3
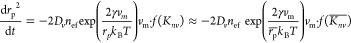
9where the transient quantities in the right-hand side of eq 9 have been approximated by the time-averaged quantifies, and note that *n*_∞_ = 0. The flat surface equilibrium concentration of glycerol vapor was hence obtained by integrating eq 9 from the inlet to the outlet of the DMA
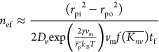
10where  was calculated based on nominal radius  converted from the nominal mobility  using eq 5a, *r*_pi_ and *r*_po_ were converted from *Z*_pi_ and *Z*_po_, respectively, based on eq 8a. All other material properties of the glycerol nanoparticle in eq 10 are summarized in the Supporting Information. The glycerol flat surface vapor pressure was then obtained based on the kinetic relationship: *P*_ef_ = *n*_ef_*k*_B_*T*. Figure 3 shows the results at eight different operation conditions compared with the data from the Chemical Engineering and Materials Research Information Center (CHERIC, 2015, see Eq. SI 3.1 of Wright et al.^17^ for the expression). Note that unlike the case of temporal size profiles, deviations show up majorly at low temperatures: rather than the absolute errors discussed previously, deviations here are majorly caused by the relative error between the true value and deduced value for the difference between nanoparticle sizes at the inlet and outlet of the DMA, i.e., *r*_pi_^2^ – *r*_po_^2^. The value of *r*_pi_^2^ – *r*_po_^2^ is comparable to experimental uncertainties at low temperatures, and hence any small experimental disturbances can lead to notable errors in the results. There are overall good agreements except for a few data points at these low temperatures. In atmospheric processes, a related complexity is that as the vapor compounds condense onto the particle phase, they may undergo reactions (e.g., proton transfers^36^) such that the resulting particle phase compounds are not representatives of the vapor compounds originally condensed. Particle phase processing may change, e.g., the vapor pressure of the particle phase compounds, which may lead to unexpected dynamics and hence biases in the particle dynamics modeling. It is thereby of importance to measure the vapor pressure of the particle phase directly. Such measurements have been very challenging, but our results here suggest a feasible methodology for realizing such measurements. However, it may be noted that vapor pressures deduced from nanoparticle evaporation rates are always subjected to uncertainties due to the assumption on (or precision of) the nanoparticle surface tension, mass accommodation coefficient, diffusion coefficient of the evaporating vapor, etc. This is also an inevitable issue faced by vapor pressure retrievals from the prevalent tandem DMA setups.^37^ The merit of this work is that it offers a way to directly measure the temporal properties of nanoparticles, such as the temporal nanoparticle size, growth/shrinkage rate, and curved surface vapor pressure from a single DMA scan of the evaporating nanoparticle in flight in the device. However, in conventional tandem setups, the two DMAs are placed at the inlet and outlet of an evaporation flowtube, and hence only sizes before and after the evaporation are measured to deduce the flat surface vapor pressure. Because results are obtained from examining the complete size-changing process in situ, the experimental uncertainties from our approach are expected to be much smaller. This is attributed to the analysis of the nanoparticle trajectory in the flow and electric field inside the DMA.

There are several other practical issues that are worth mentioning. First, atmospheric particles are typically mixtures. Though eq 7 may be invoked as an approximation for particles composed of mixed species, its precision merits further scrutiny. Second, the measurable range of vapor pressures for this approach is limited by the particle residence time in the DMA; with common residence time values 0.1–10 s, this range is approximately 10^–3^–10^–1^ Pa by assuming the vapor diffusion coefficient close to that of the glycerol. Particles with high vapor pressures can evaporate in short time intervals, e.g., the order of milliseconds for water nanoparticles. Such time scales are beyond the scope of the DMA discussed in this work. Alternative devices with smaller residence times^38−40^ may be implemented to extend the scope of the methodology, which requires further experimental demonstrations. Third, the buildup of vapors in the DMA classification region could inhibit the evaporation of nanoparticles, leading to potential errors in the theoretical prediction. According to the analysis of Wright et al., the evaporating nanoparticle can always be assumed to be surrounded by dry and clean sheath flow because the nanoparticle’s radial velocity is slightly larger than the diffusion velocity of the evaporated vapor. Their conclusion hence eliminates the issue of vapor buildup.

## Conclusions

The generality of this approach lies in the fact that the requirement on the size-changing particle due to the electric and flow field inside the DMA (i.e., eq 2) is universally applicable. Extension to a different scenario simply requires identifying the requirement from the involved physical/chemical process. More generally, if means could be developed to visualize the complete nanoparticle trajectory, then ideally, any size-change processes could be measured in situ by a “DMA-like device” without any prior knowledge of the size-change process itself. These concepts could facilitate the development of new instruments for characterizing rapid and continuous nanoparticle size-change processes due to condensation, evaporation, chemical reactions, etc., which can assist in understanding the evolution of aerosol systems, where these processes are the driving forces.

## Supporting Information Available

The theoretical derivations for operations in the free molecular and continuum regimes, numerical simulation of the evaporation of a free glycerol nanoparticle and related material properties, effects of the approximations made in eq 6b, nominal nanoparticle sizes and nanoparticle sizes at the DMA outlet at different operation conditions, temporal evaporation fluxes, and influence of the diffusion coefficient are provided in the Supporting Information (PDF)
