# Supplementary material for: Electrical Mobility as an Indicator for Flexibly Deducing the Kinetics of Nanoparticle Evaporation
Source: J Phys Chem C Nanomater Interfaces. 2022 May 11;126(20):8794–800. doi: 10.1021/acs.jpcc.2c02858 (PMC9150095; doi:10.1021/acs.jpcc.2c02858)
Supplement: Supplementary file 1 — jp2c02858_si_001.pdf [file jp2c02858_si_001.pdf]

Electrical Mobility as an Indicator for Flexibly Deducing the Kinetics of Nanoparticle Evaporation

Huan Yang*, Dian Ding, Aurora Skyttä, Runlong Cai, Markku Kulmala, and Juha Kangasluoma

Institute for Atmospheric and Earth System Research/Physics, University of Helsinki, FI-00014 Helsinki, Finland;

*Email: huan.yang@helsinki.fi

Supporting Information

Contents:

- Theoretical derivations for operations in the free molecular rand continuum regime
- Simulation of the evaporation of a free glycerol nanoparticle and related material properties
- Effects of the approximations made in Eq. 6b of the main text
- Nominal nanoparticle sizes and sizes at the DMA outlet at different operation conditions
- Temporal evaporation fluxes
- Influence of the diffusion coefficient

Theoretical derivations for operations in the free molecular and continuum regime

The derivation of the glycerol nanoparticle sizes at the DMA outlet based on measured nominal sizes for evaporation in the free molecular and continuum regime follows a similar procedure as that of the transition regime. Here, we show the theoretical methods for the free molecular and continuum regimes separately. The same set of notations are used as that of the main text.

Continuum regime. The flux in the continuum regime is:

$$J = 4\pi r_{\mathrm{p}} D_{\mathrm{v}}(n_{\infty} - n_{\mathrm{e}}), \tag{S1a}$$

with,

$$n_{\mathrm{e}} = n_{\mathrm{ef}}(T)\exp\left(\frac{2\gamma v_{\mathrm{m}}}{r_{\mathrm{p}} k_{\mathrm{B}} T}\right). \tag{S1b}$$

The relationship between the radius and mobility is:

$$Z_{\mathrm{p}} = \frac{ie}{6\pi\mu r_{\mathrm{p}}}. \tag{S2}$$

Substituting Eqs. S1a – S2 into Eq. 3 in the main text, one obtains:

$$\mathrm{d}{Z_{\mathrm{p}}}^{-2} = C\mathrm{d}t, \tag{S3a}$$

with,

$$C \equiv 2\left(\frac{ie}{6\pi\mu}\right)^{-2} D_{\mathrm{v}}(n_{\infty} - n_{\mathrm{e}})v_{\mathrm{m}}, \tag{S3b}$$

the solution of which is:

$$Z_{\mathrm{p}}(t) = \frac{1}{\left(Ct + Z_{\mathrm{pi}}{}^{-2}\right)^{1/2}}. \tag{S4}$$

Substitution of Eq. S4 into Eq. 2 in the main text yields:

$$Z_{\mathrm{po}} = Z_{\mathrm{p}}(t_{\mathrm{f}}) = \frac{1}{\frac{4\pi VL}{Q_{\mathrm{sh}} \ln(r_{\mathrm{i}}/r_{\mathrm{o}})} - \frac{1}{Z_{\mathrm{pi}}}} = \left(2\overline{Z_{\mathrm{p}}}^{-1} - Z_{\mathrm{pi}}{}^{-1}\right)^{-1}, \tag{S5a}$$

$$C = \frac{\left[\frac{4\pi VLZ_{\mathrm{pi}}}{Q_{\mathrm{sh}} \ln(r_{\mathrm{i}}/r_{\mathrm{o}})} - 1\right]^2 - 1}{Z_{\mathrm{pi}}{}^2 t_{\mathrm{f}}} = \left[\left(2\overline{Z_{\mathrm{p}}}^{-1} Z_{\mathrm{pi}} - 1\right)^2 - 1\right] Z_{\mathrm{pi}}{}^{-2} t_{\mathrm{f}}{}^{-1}. \tag{S5b}$$

Note that this result is the same as the result in the transition regime because of the form for the expression of corresponding fluxes (Eq. S1a and Eq. 4a). But Eqs. S5a and S5b should have higher precisions than Eqs. 8a and 8b when invoked in the continuum regime because one does not resort to the assumption that $f(K_{n\mathrm{v}})/\zeta^2(K_{n\mathrm{g}}) \approx f(\overline{K_{n\mathrm{v}}})/\zeta^2(\overline{K_{n\mathrm{g}}})$ in reaching Eqs. S5a and S5b.

Free molecular regime. The flux in the free molecular regime is:

$$J = \alpha\pi r_{\mathrm{p}}{}^2 c_{\mathrm{v}} (n_\infty - n_{\mathrm{e}}), \tag{S6a}$$

with

$$n_{\mathrm{e}} = n_{\mathrm{ef}}(T) \exp\left(\frac{2\gamma v_{\mathrm{m}}}{r_{\mathrm{p}} k_{\mathrm{B}} T}\right), \tag{S6b}$$

and

$$c_{\mathrm{v}} = \sqrt{\frac{8k_{\mathrm{B}}T}{\pi m_{\mathrm{v}}}}, \tag{S6c}$$

being the mean thermal speed of vapor molecules, where m_{v} is the vapor molecular mass. The relationship between the radius and mobility is:

$$z_{\mathrm{p}} = \frac{3ie}{4\pi\xi m_{\mathrm{g}} n_{\mathrm{g}} c_{\mathrm{g}} r_{\mathrm{p}}^{2}}, \quad \text{(S7)}$$

where, $\xi = 1.36$ is the momentum transfer accommodation coefficient[1], m_{g} is the background gas molecular weight, n_{g} is the background gas concentration, and c_{g} and is the background gas mean thermal speed. Substituting Eqs. S6a – S7 into Eq. 3 in the main text, one obtains:

$$\mathrm{d}Z_{\mathrm{p}}^{-1/2} = C\mathrm{d}t, \quad \text{(S8a)}$$

with

$$C \equiv \frac{1}{4}\left(\frac{3ie}{4\pi\xi m_{\mathrm{g}} n_{\mathrm{g}} c_{\mathrm{g}}}\right)^{-1/2} \alpha c_{\mathrm{v}}(n_{\infty} - n_{\mathrm{e}}) v_{\mathrm{m}}, \quad \text{(S8b)}$$

the solution of which is:

$$Z_{\mathrm{p}}(t) = \frac{1}{\left(Ct + Z_{\mathrm{pi}}^{-1/2}\right)^{2}}. \quad \text{(S9)}$$

Substitution of Eq. S9 into Eq. 2 in the main text yields:

$$Z_{\mathrm{po}} = Z_{\mathrm{p}}(t_{\mathrm{f}}) = Z_{\mathrm{pi}}^{-1}\left[\frac{Q_{\mathrm{sh}} \ln(r_{\mathrm{i}}/r_{\mathrm{o}})}{2\pi VL}\right]^{2} = Z_{\mathrm{pi}}^{-1}\overline{Z_{\mathrm{p}}}^{2}, \quad \text{(S10a)}$$

$$C = \left[\frac{2\pi VL}{Q_{\mathrm{sh}} \ln(r_{\mathrm{i}}/r_{\mathrm{o}})}\right]\frac{Z_{\mathrm{pi}}^{1/2}}{t_{\mathrm{f}}} - \frac{Z_{\mathrm{pi}}^{-1/2}}{t_{\mathrm{f}}} = \left(\overline{Z_{\mathrm{p}}}^{-1} Z_{\mathrm{pi}} - 1\right) Z_{\mathrm{pi}}^{-1/2} t_{\mathrm{f}}^{-1}. \quad \text{(S10b)}$$

Simulation of the evaporation of a free glycerol nanoparticle and related material properties

At each time step, the evaporation flux J was updated based on Eqs. 4a - 4c in the main text, fed with the glycerol nanoparticle radius from the previous time step. The glycerol nanoparticle radius at the present time step was then updated with Eq. 3 in the main text with the evaporation flux J at the present time step. This procedure was repeated for a time period equaling the residence time of the glycerol nanoparticle in the DMA, i.e. t_{f}. Thus, the temporal glycerol

nanoparticle radius profile $r_\mathrm{p}(t)$ and the (equivalent) radius at the DMA outlet $r_\mathrm{po} = r_\mathrm{p}(t_\mathrm{f})$ were obtained. The nominal glycerol nanoparticle radius in the numerical simulation was obtained by averaging the glycerol nanoparticle radius at every simulated time step, i.e.: $\overline{r_\mathrm{p}} = \left[\sum_{i=1}^{n} r_\mathrm{p}(t_i)\right]/n$ where i represents the time step and n is the total number of time steps. These radii were converted to their mobility counterparts $Z_\mathrm{p}(t)$, $\overline{Z_\mathrm{p}}$, and Z_po with Eq. 5a in the main text.

For glycerol: the diffusion coefficient was estimated with[2] $D_\mathrm{v} = D_\mathrm{v,298} \times (T/298)^{1.75}$ where $D_\mathrm{v,298}$ is the diffusion coefficient of glycerol at 298 K, taken as[3] $D_\mathrm{v,298} = 7.63 \times 10^{-6}$ m^2 s^{-1}, the mean free path of the vapor was evaluated with the kinetic relationship $\lambda_\mathrm{v} = 3D_\mathrm{v}/c_\mathrm{v}$, the mean free path of the background gas was evaluated based on $\lambda_\mathrm{g} = k_\mathrm{B}T/\left(\sqrt{2}\pi P_\mathrm{g} {d_\mathrm{g}}^2\right)$ with $P_\mathrm{g} \approx 1.013 \times 10^5$ Pa being the ambient pressure and $d_\mathrm{g} \approx 0.3$ nm, surface tension of the vapor was taken as[4] $\gamma = 0.07$ N m^{-1}, the latent heat was assume to be constant $L = 1.52 \times 10^{-19}$ J/#, molecular volume of glycerol was estimated with $v_\mathrm{m} \approx m_\mathrm{v}/\rho_\mathrm{v}$ where $m_\mathrm{v} = 0.0921/\mathrm{NA}$ kg is the vapor monomer molecular weight with NA being the Avogadro's number and $\rho_\mathrm{v} \approx 1260$ kg m^{-3} is the glycerol density, the mass accommodation coefficient was assumed to be unit $\alpha = 1$ for the solid line and $0.6 < \alpha < 1$ for the shadowed region in Fig. 2, and the sheath flow was assumed to be dry air (in consistent with the experimental conditions) such that $n_\infty = 0$.

For water: properties are obtained online at https://www.engineeringtoolbox.com/.

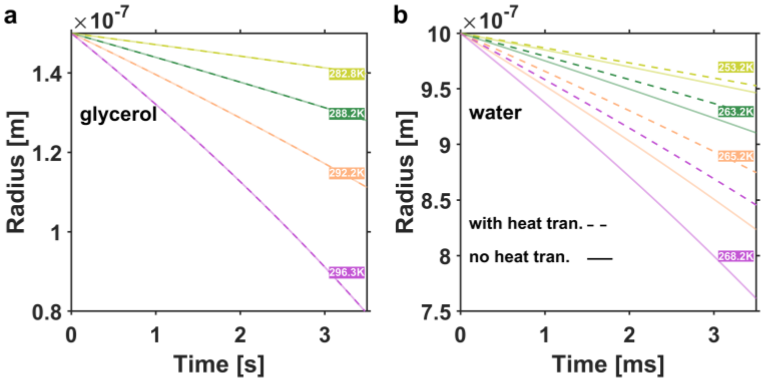

Figure S1. Size evolution of free glycerol and water particles at selected temperatures with or without heat transfer.

Effects of the approximations made in Eq. 6b of the main text

The simulation procedure for obtaining nanoparticle mobilities at the DMA outlet (solid line) and nominal mobilities (dotted line) in the figure has been described in the previous section. The simulations did not include the constant variable *C* approximations made in Eq. 6. On the other hand, the hollow circles denote nanoparticle mobilities at the DMA outlet calculated based on Eq. 8a with the simulated nominal mobility (dotted line) as the input, where the constant variable *C* approximations was included. The agreement between the solid line and hollow circles indicate that errors introduced by approximations in Eq. 6 are negligible.

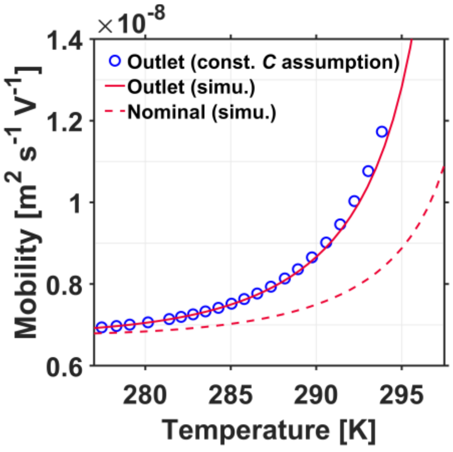

Figure S2. Effect of the approximations made in Eq. 6 of main text ($Q_{\mathrm{sh}} = 26.48$ L min^{-1} and $r_{\mathrm{pi}} = 151$ nm)

Nominal nanoparticle sizes and sizes at the DMA outlet at different operation conditions

Fig. S3 shows the nominal radius from measurements of Wright et al.[4] and simulation, radius at the DMA outlet from simulation and Eq. 8a in the main text for the eight different operation conditions. In all cases, good agreements between the measurements and simulations (or predictions of Eq. 8 and simulations) can be seen, which validates the proposed approach.

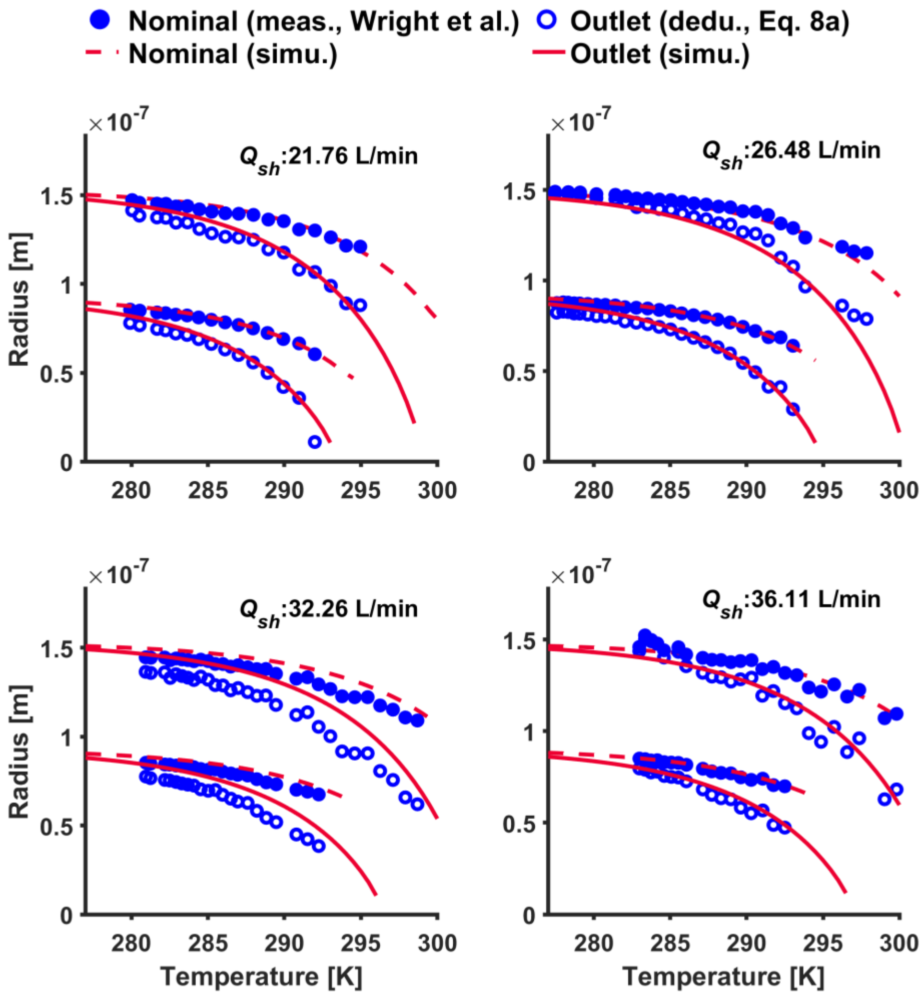

Figure S3. The glycerol nanoparticle nominal radius and radius at the DMA outlet at eight different operation conditions with (a) $Q_{\mathrm{sh}} = 21.76$ L min^{-1} , $r_{\mathrm{pi}} = 153$ nm (upper), $r_{\mathrm{pi}} = 93$ nm (lower), (b) $Q_{\mathrm{sh}} = 26.48$ L min^{-1}, $r_{\mathrm{pi}} = 151$ nm (upper), $r_{\mathrm{pi}} = 93$ nm (lower), (c) $Q_{\mathrm{sh}} = 32.26$ L min^{-1}, $r_{\mathrm{pi}} = 153$ nm (upper), $r_{\mathrm{pi}} = 93$ nm (lower), and (d) $Q_{\mathrm{sh}} = 36.11$ L min^{-1}, $r_{\mathrm{pi}} = 148$ nm (upper), $r_{\mathrm{pi}} = 90$ nm (lower).

Temporal evaporation fluxes

Fig. S4 shows the temporal evolution of the fluxes at six selected system temperatures for the case of $Q_{\mathrm{sh}} = 26.48$ L min^{-1} and $r_{\mathrm{pi}} = 151$ nm. These fluxes were calculated by substituting the temporal radii into Eq. 4a in the main text; for the numerical results, the temporal radii were evaluated from iterating Eq. 3 with assuming a free evaporating nanoparticle, where the used material properties are summarized in the previous section, while for the deduced results, the

temporal radii were converted (based on Eq. 5a) from the expression of the temporal mobilities (Eqs. 7 and 8b). Overall, good agreements between the simulated fluxes and the deduced fluxes can be observed. Only slight deviations can be seen at the temperature of 296.3 K, which is caused by the assumptions made in the theoretical derivations (assuming C to be a constant in Eq. 6b).

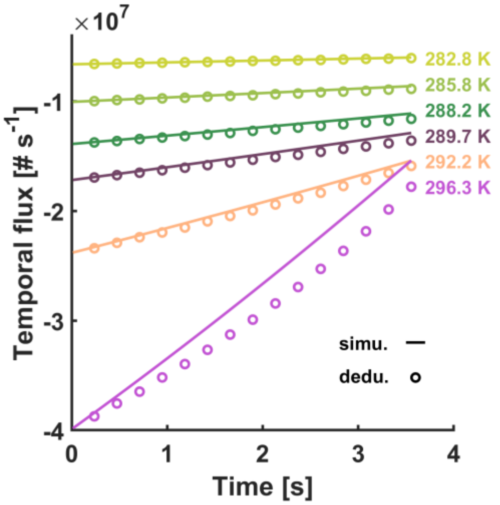

Figure S4. Temporal evaporation fluxes at different system temperatures for the case of $Q_{\mathrm{sh}} = 26.48$ L min^{-1} and $r_{\mathrm{pi}} = 151$ nm.

Influence of the diffusion coefficient

The diffusion coefficient essentially determines the flux of evaporation, thus it is helpful to check its influence on the calculations. The value of the diffusion coefficient influences the numerical simulated results of the glycerol nanoparticle radius at the DMA outlet but will not influence the theoretically deduced values, as the latter is lumped in the constant C which is derived from the operation conditions of the device and the experimentally measured nominal radius. We increased and decreased the value of the diffusion coefficient used in the main text by 50% respectively, to mimic possible errors in the estimation. The results for the simulated glycerol nanoparticle radius at the DMA outlet are shown in Fig. S5 for the case of $Q_{\mathrm{sh}} = 26.48$ L min^{-1}

and $r_{\mathrm{pi}} = 151$ nm. Overall, this range leads to results that overlap well with the deduced results, indicating that the diffusion coefficient value adopted is reasonable.

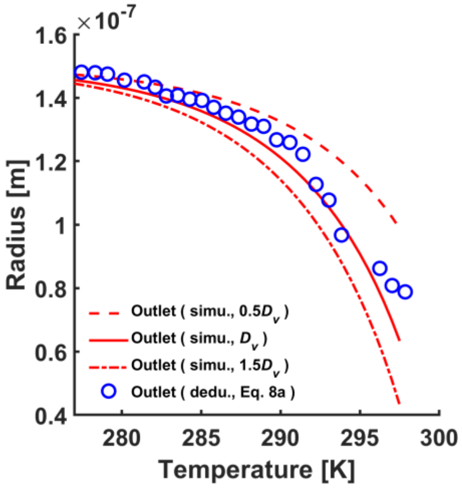

Figure S5. Influence of the diffusion coefficient on the simulated glycerol nanoparticle radius at the DMA outlet for the case of $Q_{\mathrm{sh}} = 26.48$ L min^{-1} and $r_{\mathrm{pi}} = 151$ nm.

REFERENCES

[1] Larriba, C.; Hogan Jr, C. J. Ion Mobilities in Diatomic Gases: Measurement Versus Prediction with Non-Specular Scattering Models. *J. Phys. Chem. A* **2013**, *117*, 3887-3901.

[2] Tang, M.; Shiraiwa, M.; Pöschl, U.; Cox, R.; Kalberer, M. Compilation and Evaluation of Gas Phase Diffusion Coefficients of Reactive Trace Gases in the Atmosphere: Volume 2. Diffusivities of Organic Compounds, Pressure-Normalised Mean Free Paths, and Average Knudsen Numbers for Gas Uptake Calculations. *Atmos. Chem. Phys.* **2015**, *15*, 5585-5598.

[3] Bird, R. B.; Stewart, W. E.; Lightfoot, E. N. *Transport Phenomena*; John Wiley & Sons, 2006.

[4] Wright, T. P.; Song, C.; Sears, S.; Petters, M. D. Thermodynamic and Kinetic Behavior of Glycerol Aerosol. *Aerosol Sci. Technol.* **2016**, *50*, 1385-1396.
